# Huge chondromyxoid fibroma of the right iliac wing with tremendous soft tissue extensions

**DOI:** 10.1259/bjrcr.20170014

**Published:** 2017-10-21

**Authors:** Hosameldeen Mostafa Ali

**Affiliations:** Department of Radiology, Benha University, Benha, Egypt

## Abstract

This report describes a huge chondromyxoid fibroma (CMF) that developed in the right iliac crest and wing. The tumour is rare, perhaps the rarest of all bone tumours, and its occurrence in the iliac crest and wing of a 63-year-old male is extraordinarily uncommon. The patient complained of gradual onset of right groin pain over a period of more than 2 years and low back pain and tender swelling of the right gluteal region over a period of another 1 year. Conventional radiography of the lumbar spine and pelvis revealed a large osteolytic lesion of the right iliac crest and wing associated with mild levoscoliosis. MRI of the pelvis revealed a huge well-defined lesion arising from the right iliac crest and wing and extending to the right paraspinal region, false pelvis and right gluteal region and displacing rather than invading the surrounding structures. The patient underwent surgery, and the mass was totally removed. The clinical manifestations, imaging findings and surgical treatment of the lesion are discussed.

## Background

Chondromyxoid fibroma (CMF) is a rare, benign tumour that resembles cartilage, initially arising in the cortex of affected bones (most commonly the lower limbs). Its documented incidence is less than 1% of all primary bone tumours (about 2% of all benign bone tumours) with males and females being equally affected. It has a potential for regional enlargement towards the local tissues. It consists of immature myxoid mesenchymal tissue with features of primitive cartilaginous differentiation. Patients most commonly affected are in their second or third decade of life. An additional peak of incidence is observed between 50 and 70 years of age.^1^

The imaging features of CMF in the radiological literature mostly apply to lesions arising from the long bones. The current report aims to describe the imaging features and behaviour of CMF arising from the flat bones of the pelvis and to differentiate it from more aggressive lesions such as chondrosarcoma. The MR criteria of CMF are the peripheral low to the intermediate signal band and the central hyperintense signal on *T*_2_ weighted images, and the diffuse low signal on *T*_1_ weighted images. CMF of flat bones of the pelvis could reach a huge size, and it could extend to the paraspinal region, the pelvis and the gluteal region, displacing and not invading the surrounding structures.

CMF with atypical radiographic findings may mimic more common and more aggressive tumours. The differential diagnosis of CMF includes a variety of bone lesions. CMF lesions at unusual sites or age groups could lead to misinterpretation and unnecessary investigations.^2^

## Case report

A 63-year-old male presented first with right groin pain and back pain over a period of more than 2 years and received analgesics, which did not help much. A year later, he started to feel pain and swelling in the right gluteal region.

**Figure 1.**
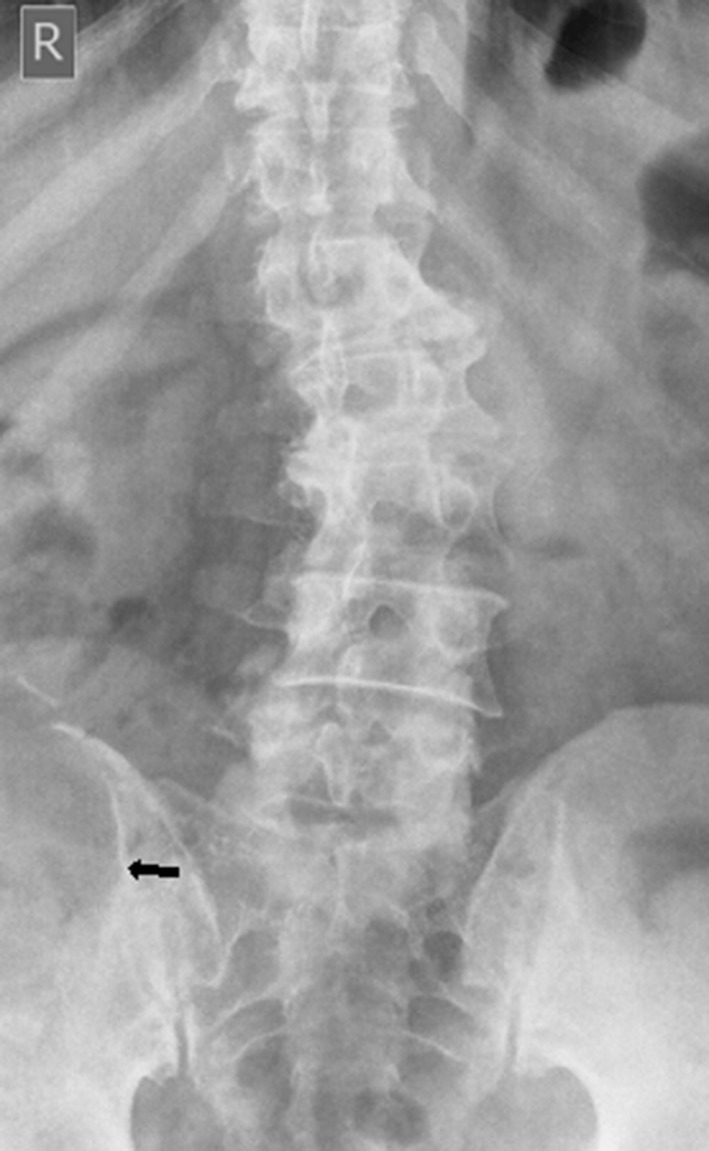
Pain x-ray lumbosacral spine anteroposterior projection revealed mild levoscoliosis and partially visualized osteolytic lesion (black arrow) of right iliac crest and wing.

Conventional radiography of the lumbar spine showed mild left convex scoliosis and a partially elicited right iliac wing osteolytic lesion (Figure 1).

**Figure 2.**
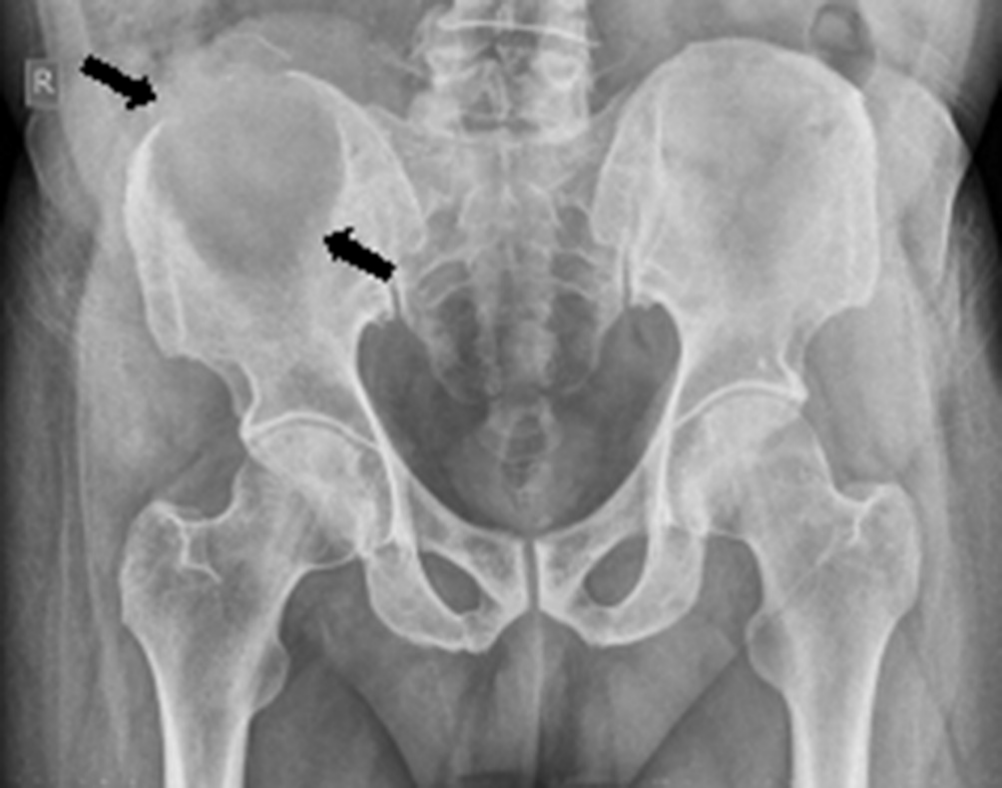
Plain X-ray pelvis anteroposterior projection revealed a large osteolytic lesion (black arrows) with sclerotic margins involving the right iliac crest and wing. No obvious matrix gross calcifications were seen.

Conventional radiography of the pelvis revealed a large osteolytic lesion involving the right iliac crest and wing with sclerotic margins and no obvious dominant gross matrix calcifications (Figure 2).

**Figure 3.**
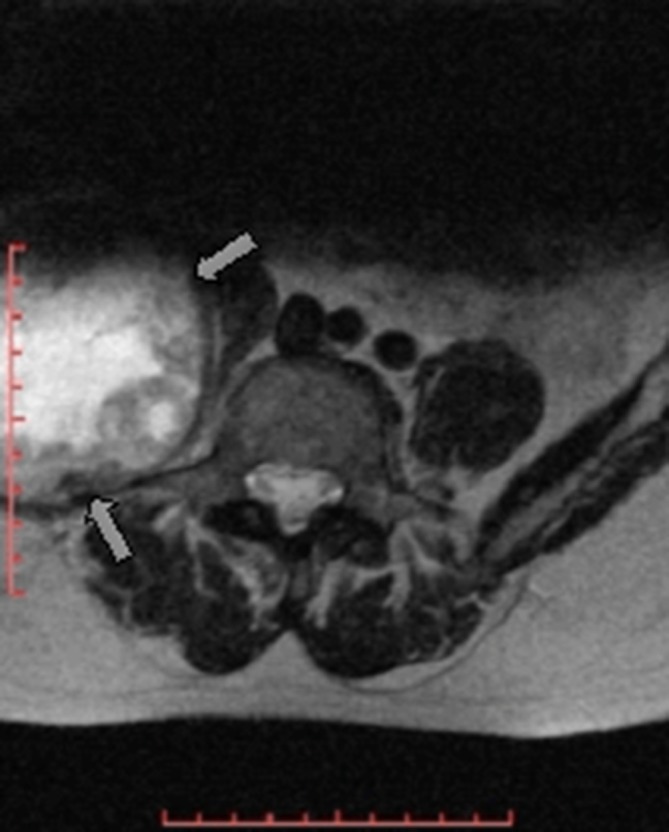
Axial *T*_2_ MR image of the lumbar spine revealed partially visualized heterogeneous hyperintense right paraspinal lesion (white arrow).

**Figure 4.**
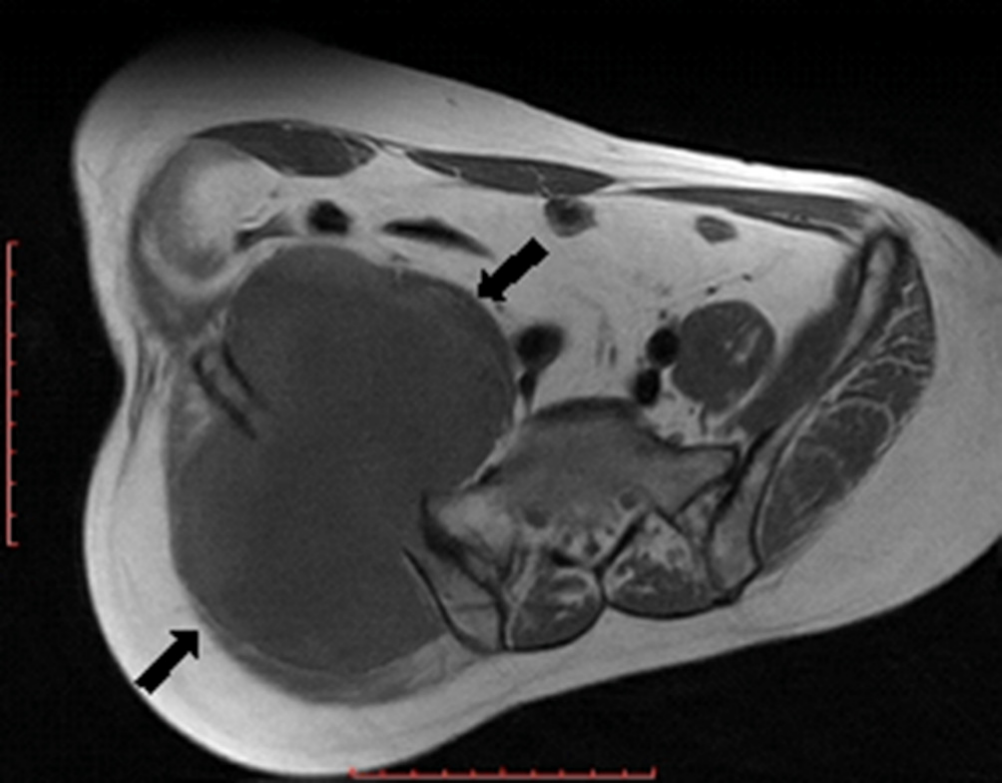
Axial *T*_1_ fast spin echo MR image showed huge homogenous hypointense lesion (black arrows) involving the right iliac wing and extending to the false pelvis and right gluteal region. The lesion displaced and attenuated the right iliopsoas and gluteal muscles. No evidence of invasion or encasement of right iliac vessels was seen.

**Figure 5.**
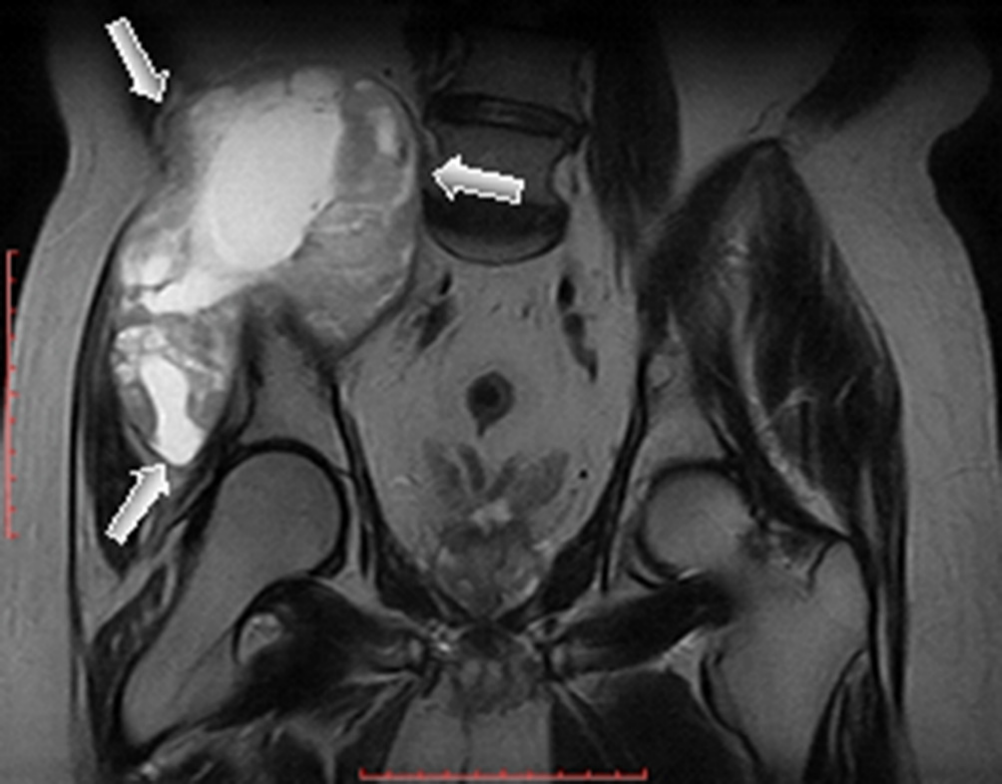
Coronal *T*_2_ fast spin echo MR image showed heterogeneous, predominantly hyperintense lesion (white arrows) involving the right iliac wing and extending to the false pelvis, approaching the right paraspinal region and right gluteal region.

**Figure 6.**
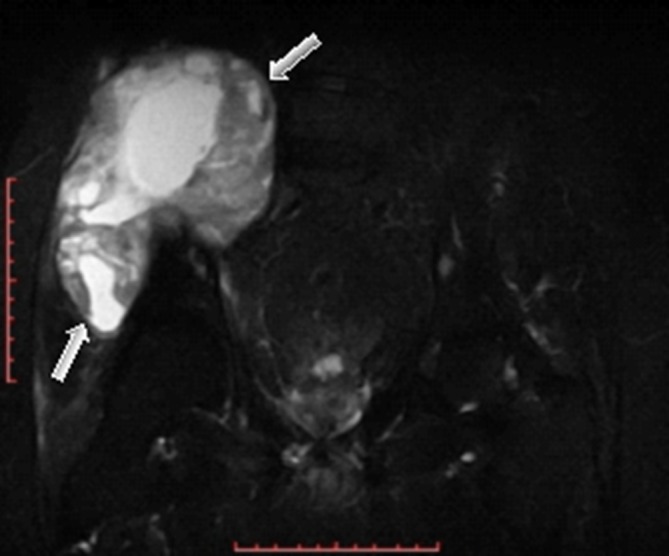
Coronal short tau inversion-recovery MR image showed heterogeneous hyperintense lesion involving the right iliac wing and extending to the false pelvis, approaching the right paraspinal region and right gluteal region.

**Figure 7.**
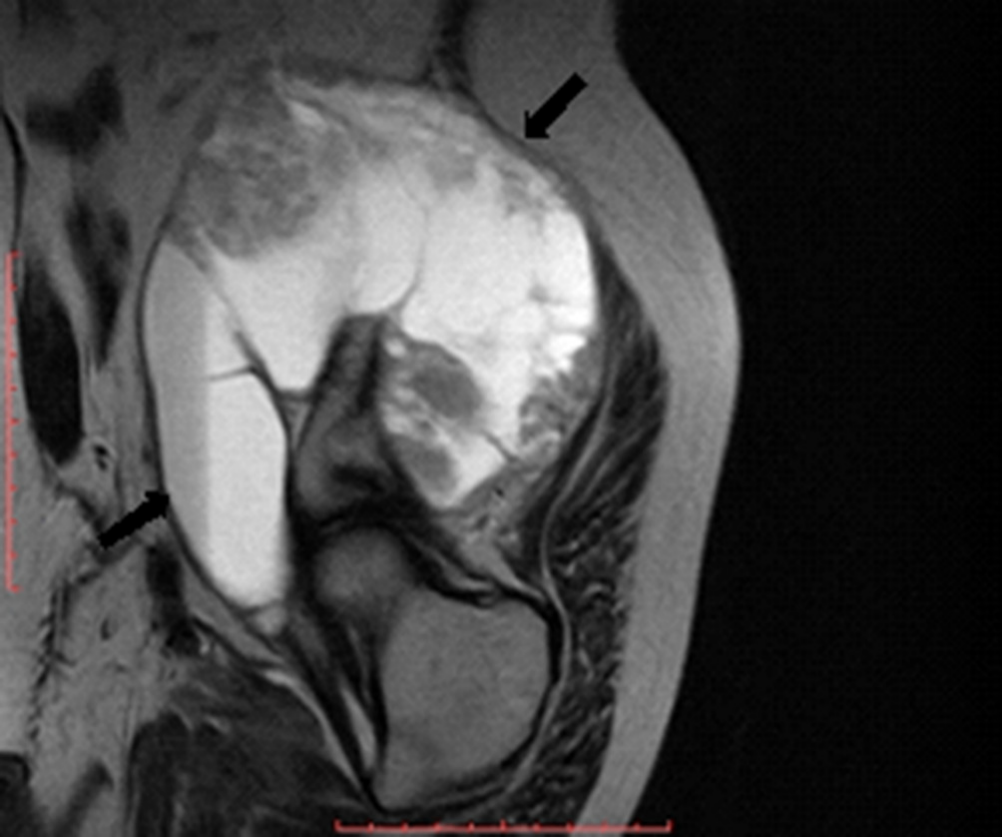
Sagittal *T*_2_ fast spin echo MR image showed heterogeneous hyperintense lesion (black arrows) involving the right iliac wing and extending to the false pelvis, approaching the right paraspinal region and right gluteal region.

MR examination of the pelvis elicited a huge (about 176 × 129 × 94 mm) mass arising from the superior aspect of the right iliac wing, which is generally *T*_1_ homogenous hypointense and *T*_2_ heterogeneous hyperintense signal with internal dominant fluid-equivalent signal and peripheral nodular hypointense margin. The mass is saddle shaped, overhanging the right iliac crest with a pelvic (internal) limb displacing and attenuating the right iliopsoas muscle without invasion of the iliac vessels and gluteal (external) limb extending into the gluteal region between the gluteal medius and minimus muscles. No evidence of invasion or encasement of the surrounding structures or adjacent iliac vessels was observed, and there was no defined regional pelvic lymphadenopathy. The whole lesion was surgically excised. There was no defined invasion of the surrounding adjacent soft tissues of the pelvis or the right gluteal region (Figures 3–7).

## Discussion

CMF is a rare benign bone tumour accounting for about less than 0.5% of all bone tumours. It is a very slow-growing tumour and usually presents with a large mass. The matrix is predominantly cartilaginous with varying chondroid, fibrous and myxoid elements.

All the bones could be affected, however; the most common sites of involvement include the metaphysis of long bones. Involvement of lower limbs is 5-fold more common than that of the upper limbs. Involvement of the small bones of the feet is 5-fold greater than that of the hands.^3^

CMF has a predilection to occur in the knee region, especially in the proximal tibial metaphysis. Only five cases of chondromyxoid fibroma of the pelvis have been reported.^4^

Pelvic bones are a rare location for CMF. Iliac wings are an unusual site for this rare benign bone tumour of cartilaginous origin. A wide variety of benign and malignantlesions can arise from the pelvic bones. The differentiation between such lesions mainly relies on the patient’s age, gender, the number of lesions, type of matrix and the presence or absence of invasion of the surrounding structures and regional or remote lymphadenopathy. Conventional radiography and CT are necessary for matrix characterization, particularly, detection of mineralization. MRI is helpful for further characterization and radiological differential diagnosis.

Small CMFs are usually asymptomatic. Large lesions might cause local pain, swelling or distortion of bone.^5^

The radiological appearance of CMF is not specific and may often mimic more common bone lesions, particularly when it arises from an unusual site such as the iliac wing. These radiological findings are variable, depending on the anatomical site of the lesion. CMF could be considered in the differential diagnosis in cases with well-defined multilocular lesions with sclerotic borders and absent periosteal reaction. An expansile ovoid lesion with a radiolucent centre is the usual radiographic pattern. Well-defined sclerotic margins, internal septations and a bulging thinned overlying cortex are frequent CMF findings.^6^

The conventional radiographic features of chondromyxoid fibroma are expansile, lytic, eccentric and well demarcated. Calcification might exist with a variable incidence ranging from 11 to 14%.^3^

Internal septations might exist. The presence, pattern and distribution of matrix calcifications are better identified and characterized by CT, not conventional radiography.^7^

Microscopically, there is a higher incidence of calcifications up to 34%, much more than that detected on conventional radiography or CT. Calcifications are more prevalent in older patients and in flat bones.^4^

The tumour typically demonstrates heterogeneous low signal intensity on *T*_1_ weighted images. Peripheral low to intermediate margin and central heterogeneous bubbly high signal intensity on *T*_2_ weighted and short tau inversion-recovery images with predominant fluid-equivalent signal are seen.^8^

Heterogeneous matrix enhancement achieved post-contrast injection is typically more marked at the vascular borders of the tumour. Heterogeneity is due to the existence of cystic and/or haemorrhagic components of the lesion.^9^

MR features of the CMF still are nonspecific, yet considered the mainstay for preoperative planning.^10^

A single imaging modality alone is insufficient for making a diagnosis of CMF. Combination of imaging modalities such as conventional radiography, MR and/or CT is recommended to characterize the lesion and narrow the spectrum of differential diagnosis.

Most criteria of CMF in the imaging literature are applicable to the more common sites of origin of long bones about the knee region. Few cases in the imaging literature described the CMF of flat bones, namely the iliac wings.

The imaging differential diagnosis of CMF should consider the site of origin of the lesion and includes a variety of bone lesions such as cartilaginous tumours (chondroblastoma, giant cell tumour, enchondroma and low-grade chondrosarcoma), osteogenic tumours (osteoid osteoma and osteoblastoma), fibrous tumours (non-ossifying fibroma), non-neoplastic reactive lesions (aneurysmal bone cyst) and other benign lesions (eosinophilic granuloma, giant cells tumour). Malignant tumours (multiple myeloma, metastasis, chondrosarcoma, osteoblastoma and chordoma) must also be considered in certain cases.^11^

An aneurysmal bone cyst inside a CMF is rare but possible.^12^

Malignant transformation of CMF to chondrosarcoma was seen in several cases, yet none of those cases were sufficiently documented.^13^

Some cases of CMF showed partial features of chondrosarcoma without metastasis. The hypothesis of primary malignant CMF was rejected.^14^

The first-year postoperative recurrence rate could reach up to 11%. Incomplete surgical excision carries the risk of higher recurrence rates (up to 80%). Radiotherapy could lead to malignant transformation.^15^

Total excision with safety (tumour-free) margins is the treatment of choice for CMF. Intralesional curettage is associated with high (up to 25%) recurrence rates. Despite the benign nature of CMF, its local aggressiveness should not be underestimated.^4^

## Conclusions

CMF shows indistinctive imaging patterns in most cases. Sclerotic rim and sometimes bubbly-appearing lesions appear on conventional radiography. CT scans might reveal calcifications within the tumour that is not visible on conventional radiographs. When occurring in unusual locations or in older patients, as in the current case, careful differentiation from more serious and aggressive tumours such as chondrosarcoma is a must. The tumour typically demonstrates diffuse low signal intensity on *T*_1_ weighted images and heterogeneous, peripheral low to intermediate and central high signal intensity on *T*_2_ weighted images.

The presented case is unusual with regard to patient’s age, site of involvement, overall lesion size and extensions. The mass overhangs the iliac crest and extensively extends to the paraspinal region, pelvis and gluteal region with displacement and compression rather than the invasion of the surrounding structures.

Imaging evaluation and analysis, particularly MR, is generally useful in distinguishing CMF from other possible entities. Multimodality imaging workup is probably required to narrow the scope of differential diagnosis of CMF to overcome the indistinctive imaging features of the tumour on each single modality and to achieve an accurate estimation of the margins and soft tissue extensions and relations of the lesion. Imaging is crucial to differentiate CMF from more aggressive lesions and to plan for surgery.

## Learning points

1. CMF is a rare, very slowly growing tumour, and usually involves patients in the second and third decades of life, with a predilection to the metaphysis of long bones.
2. When occurring in unusual locations or in older patients, careful differentiation from more serious and aggressive tumours such as chondrosarcoma is crucial.
3. The helpful MR features of CMF are the peripheral intermediate signal band and the central hyperintense signal on *T*_2_ weighted images. *T*_1_ weighted images showed hypointense to intermediate signal intensity throughout the lesion.
4. Despite the benign nature of CMF, its local aggressiveness should not underestimated.
5. Multimodality imaging workup is probably needed to narrow the scope of differential diagnosis, differentiate CMF from more aggressive lesions and meet proper management planning.

## Consent

Written informed consent for the case to be published (including images, case history and data) was obtained from the patient(s) for publication of this case report, including accompanying images.

## References

[1] Budny AM, Ismail A, Osher L. Chondromyxoid fibroma. J Foot Ankle Surg 2008; 47: 153–9. 18312923 10.1053/j.jfas.2007.08.013

[2] Dürr HR, Lienemann A, Nerlich A, Stumpenhausen B, Refior HJ. Chondromyxoid fibroma of bone. Arch Orthop Trauma Surg 2000; 120: 42–7. 10653103 10.1007/pl00021214

[3] Zillmer DA, Dorfman HD. Chondromyxoid fibroma of bone: thirty-six cases with clinicopathologic correlation. Hum Pathol 1989; 20: 952–64. 2793160 10.1016/0046-8177(89)90267-0

[4] Wu CT, Inwards CY, O'Laughlin S, Rock MG, Beabout JW, Unni KK Chondromyxoid fibroma of bone: a clinicopathologic review of 278 cases. Hum Pathol 1998; 29: 438–46. 9596266 10.1016/s0046-8177(98)90058-2

[5] Erlemann R. Imaging and differential diagnosis of primary bone tumors and tumor-like lesions of the spine. Eur J Radiol 2006; 58: 48–67. 16431065 10.1016/j.ejrad.2005.12.006

[6] Erlemann R. Imaging and differential diagnosis of primary bone tumors and tumor-like lesions of the spine. Eur J Radiol 2006; 58: 48–67. 16431065 10.1016/j.ejrad.2005.12.006

[7] Hau MA, Fox EJ, Rosenberg AE, Mankin HJ Chondromyxoid fibroma of the metacarpal. Skeletal Radiol 2001; 30: 719–21. 11810171 10.1007/s002560100428

[8] Oh N, Khorsandi AS, Scherl S, Wang B, Wenig BM, Manolidis S, et al Chondromyxoid fibroma of the mastoid portion of the temporal bone: MRI and PET/CT findings and their correlation with histology. Ear Nose Throat J 2013; 92: 201–3. 23599102 10.1177/014556131309200412

[9] Soler R, Rodríguez E, Suárez I, Gayol A. Magnetic resonance imaging of chondromyxoid fibroma of the fibula. Eur J Radiol 1994; 18: 210–1. 7957292 10.1016/0720-048x(94)90336-0

[10] Murata H, Horie N, Matsui T, Akai T, Ueda H, Oshima Y, et al Clinical usefulness of thallium-201 scintigraphy and magnetic resonance imaging in the diagnosis of chondromyxoid fibroma. Ann Nucl Med 2008; 22: 221–4. 18498038 10.1007/s12149-007-0102-3

[11] Murphey MD, Walker EA, Wilson AJ, Kransdorf MJ, Temple HT, Gannon FH, et al From the archives of the AFIP: imaging of primary chondrosarcoma: radiologic-pathologic correlation. Radiographics 2003; 23: 1245–78. 12975513 10.1148/rg.235035134

[12] Dahlin DC, Unni KK. Bone tumors: general aspects and data on 8542 cases. 4th ed Springfield, IL: Charles C Thomas; 1986.

[13] Uematsu A, Coy JT, Hodges SO, Goodman RP, Brower TD Malignant chondromyxoid fibroma of the scapula. South Med J 1977; 70: 1469–71. 594803 10.1097/00007611-197712000-00029

[14] Schajowicz F. Tumors and tumorlike lesions of bone. Springer, Berlin Heidelberg, New York 1981;148–60.

[15] Gherlinzoni F, Rock M, Picci P. Chondromyxoid fibroma. The experience at the Istituto Ortopedico Rizzoli. J Bone Joint Surg Am 1983; 65: 198–204. 6337162 10.2106/00004623-198365020-00008

